# The Association of Carotid Plaque Size, Carotid Intima-Media Thickness, Resistive Index, and Pulsatility Index With Acute Ischemic Stroke

**DOI:** 10.7759/cureus.41384

**Published:** 2023-07-05

**Authors:** Ghulam Kawnayn, Humayun Kabir, Muhammad Rezeul Huq, Md. Ismail Chowdhury, Md. Shahidullah, Bonny Sadar Hoque, Mahin Binte Anwar

**Affiliations:** 1 Neurology, Combined Military Hospital, Dhaka, BGD; 2 Neurology, Bangabandhu Sheikh Mujib Medical University, Dhaka, BGD; 3 Internal Medicine, Combined Military Hospital, Dhaka, BGD; 4 Radiology, Combined Military Hospital, Dhaka, BGD

**Keywords:** resistive index (ri), carotid atherosclerosis, carotid duplex ultrasound, ischemic stroke, pulsatility index (pi), carotid intima-media thickness (cimt), carotid plaque

## Abstract

Background: Carotid atherosclerosis is an important etiological factor for ischemic stroke. Early carotid lesion detection may prevent further strokes. Doppler ultrasound measures carotid artery blood flow, intima-media thickness, stenosis, plaques, and lumen and wall changes.

Objective: The objective of this study is to determine the association of carotid plaque size (CPS), carotid intima-media thickness (CIMT), resistive index (RI), and pulsatility index (PI) with acute ischemic stroke.

Methodology: A total of 60 participants were taken, including 30 cases and 30 age- and sex-matched controls. Patients with acute ischemic stroke were included as cases and age- and sex-matched healthy volunteers were included as controls. A carotid duplex study was done in both groups, and the findings were compared.

Results: The mean age of the ischemic stroke cases was 63.33±10.79 years; more than half were aged >60 years. Male patients were 73.3% and female patients were 26.7% of the cases. Age and gender were statistically similar (p>0.05) in groups (cases and controls). The plaques were homogenous in 30% of patients, calcified in 26.7%, and mixed in 6.7%. About 36.6% of patients did not have any plaque. CPS was greater among cases than controls in the right and left internal and left common carotid arteries; however, it was not statistically significant (p>0.05). The mean CIMT was 0.79±0.10 mm on the right side and 0.90±0.17 mm on the left side among cases. CIMT was significantly higher in the cases group than in the controls (p<0.05). The mean RI was significantly greater in the left common carotid artery (CCA) among the cases than in the controls (p<0.05). Patients aged over 60 years had significantly higher RI and PI values in the left internal carotid artery (ICA) compared to the younger cases (p<0.05). Smoking history had a significant association with left CCA RI and PI values (p<0.05). However, RI, PI, CPS, and CIMT among cases were similar in different groups like diabetic, non-diabetic, hypertensive, and non-hypertensive patients (p>0.05).

Conclusion: CIMT was significantly thicker among the patients with ischemic stroke compared to the control group. RI in the left CCA was significantly greater among the stroke patients than in the control group. The age of the patient and smoking habit had an association with RI and PI values. Most of the parameters were found to be significant on the left side, suggesting carotid atherosclerosis may not be symmetrical. A large-scale further study is needed to see the association of these variables with ischemic stroke patients.

## Introduction

Stroke causes morbidity and mortality and is the second largest cause of death globally [1]. Ischemic stroke is the most common variety of stroke and constitutes an estimated 85% of all stroke cases [2]. Two main pathophysiological mechanisms are responsible for ischemic stroke: embolism and small vessel disease. In the majority of cases, emboli are formed inside the large artery or cardiac chamber [3]. Carotid atherosclerosis is the key factor leading to artery-origin emboli and is responsible for 15-20% of all ischemic stroke cases [4]. Unstable or ruptured atherosclerotic plaques in the common carotid artery (CCA) bifurcation or the proximal portion of the internal carotid artery (ICA) act as the triggering event for thrombus formation. These thrombi may dislodge and cause the occlusion of the distal arteries, resulting in the clinical features of a transient ischemic attack (TIA) or stroke [5]. As there is definitive treatment, including medical management, carotid endarterectomy, or carotid angioplasty and stenting, available for carotid atherosclerosis, it is an important reversible risk factor of ischemic stroke.

Doppler ultrasonography is used to examine carotid arteries during a cardiac cycle. It assesses intima-media thickness (IMT), stenosis, plaques, and other lumen and wall alterations. This is a safe, affordable, reliable, repeatable, and convenient method to detect carotid atherosclerosis [5,6]. The thickness between the intimal-luminal and the medial-adventitial interfaces is defined as the IMT or carotid intima-media thickness (CIMT) [7].

CIMT varies from 0.25 to 1.5 mm in healthy persons; levels over 1.0 mm are considered abnormal [8]. Increased CIMT is associated with carotid atherosclerosis and is recognized as a risk factor for cardiovascular diseases [9]. Carotid artery plaque is another cardiovascular risk factor that is closely associated with CIMT. It can be defined as a localized thickening inside the arterial lumen of at least 0.5 mm or 50% larger than the surrounding CIMT value or >1.5 mm thickness of the CIMT [10]. Recent literature reveals plaque area may be a better marker of atherosclerosis than plaque thickness or IMT [11]. The pulsatility index (PI) is defined as the difference between peak systolic and minimum diastolic velocities divided by the cardiac cycle mean velocity. PI reflects artery distal vascular resistance [12]. Stenosis of the small intracranial perforating arteries may impact the proximal artery's PI, making it a strong predictor of cerebral infarction [13]. On the other hand, a similar parameter, the arterial resistive index (RI), is related to both vascular resistance and compliance [14]. Carotid artery RI is also an important indicator of carotid atherosclerosis and is related to cardiovascular diseases [15]. In Bangladesh, little research has examined these factors in acute ischemia. The current research evaluated carotid plaque size (CPS), CIMT, RI, and PI in acute ischemic stroke patients.

## Materials and methods

This case-control study was conducted in the Department of Neurology at the Combined Military Hospital, Dhaka, from August 2019 to July 2020. Over the course of one year, 60 participants (30 in each group) were taken as the study population.

After ethical clearance from the Ethical Committee of Armed Forces Medical Services, Bangladesh (4614/35/T/DGMS/Ethi), informed written consent was taken from each patient or their attendant. Consecutive patients with an ischemic stroke fulfilling the inclusion and exclusion criteria were included as cases. Patients aged over 18 years of both sexes with acute ischemic stroke diagnosed by clinical findings (onset of symptoms not more than seven days) and CT scan/MRI of the brain who are willing to participate were included in the case group. Lesions were classified as total anterior circulation infarct (TACI), partial anterior circulation infarct (PACI), posterior circulation infarct (POCI), and lacunar infarct (LACI) according to the Bamford classification [16]. But evidence of the cardiac cause of embolization (after evaluation by routine ECG, transthoracic or transesophageal echocardiogram, Holter ECG), history of previous ischemic stroke, having a history of head injury, and evidence of intracranial hemorrhage or space-occupying lesion on the computed tomography (CT) scan of the brain were excluded from this study. On the other hand, age- and sex-matched healthy volunteers without any history of stroke, confirmed by a normal CT scan/MRI of the brain, were included in the control group. The controls were attendants of our indoor patients and volunteered in the study.

**Figure 1:**
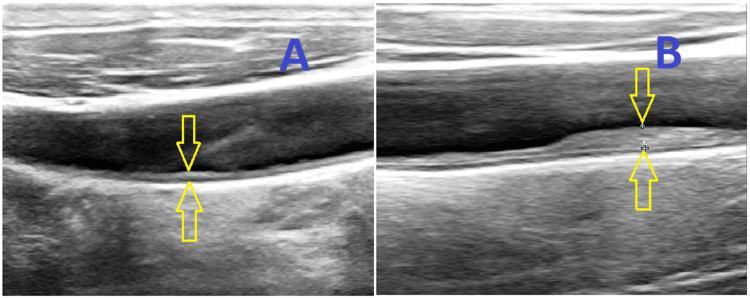
Duplex ultrasonography (B-mode image) shows the distances between the intimal-luminal (upper arrow) and the medial-adventitial interfaces (lower arrow). CIMT (A) and carotid plaque (B). CIMT: carotid intima-media thickness.

**Figure 2:**
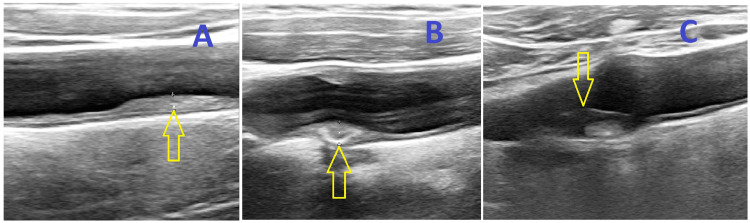
Duplex ultrasonography (B-mode image) shows different types of carotid plaques (arrows). Homogenous (A), calcified (B), and mixed (C).

Carotid plaque was defined as a focal thickening of 50% greater than the surrounding area or greater than 1.5 mm [10]. The IMT measurement was made using a Vivid 7 Pro version 7 xx Honter, Norway USG machine, with a 12 MHz linear probe in B-mode. The distances between the intimal-luminal and the medial-adventitial interfaces were recorded as IMT or carotid plaque (Figure 1). The types of carotid plaques were documented (Figure 2). All readings were taken and interpreted by the same investigator. We assessed the carotid ultrasound scan (USG) parameters irrespective of sides, whether it was ipsilateral or contralateral to the stroke. The point of measurement was taken 1 cm proximal to the carotid bulb at the site of maximum thickness, avoiding the plaque area. The ultrasound machine used had a sensitivity range of 0.1 mm, that is, each division was equivalent to 0.1 mm. RI and PI were also measured using a Vivid 7 Pro version 7 xx Honter, Norway USG machine, with a 12 MHz linear probe and color or power Doppler to help vessel localization and blood flow measurement. The RI and PI were calculated automatically by the USG machine.

We analyzed data using IBM SPSS Statistics for Windows, Version 22.0 (Released 2013; IBM Corp; Armonk, New York, United States). Continuous parameters were expressed as mean ± standard deviation (SD) and categorical parameters as percentages. The Shapiro-Wilk test of normality was done to see whether the data were normally distributed or not. To determine the difference between continuous variables, the independent sample Mann-Whitney U test and the independent samples t-test were done. To determine the association between qualitative variables, a chi-square test was done. A p-value of ≤0.05 was considered significant. The result was presented using tables, figures, charts, and textual summaries.

## Results

**Figure 3:**
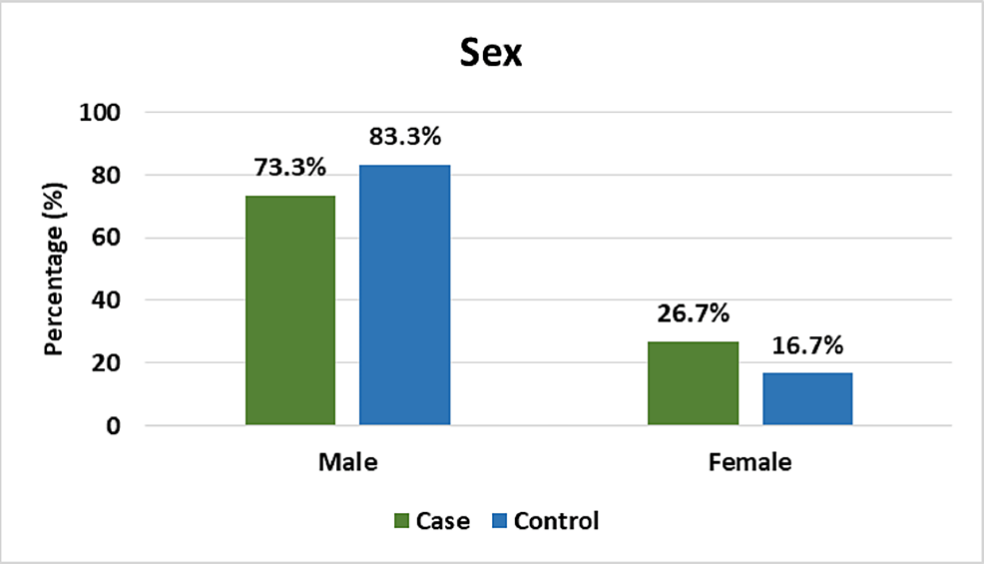
Distribution of study population according to sex (n=60).

Figure 3 shows that 73.3% of respondents were male and 26.7% were female in the case group; 83.3% were male and 16.7% were female in the control group. Both groups were statistically similar (p=0.347).

**Figure 4:**
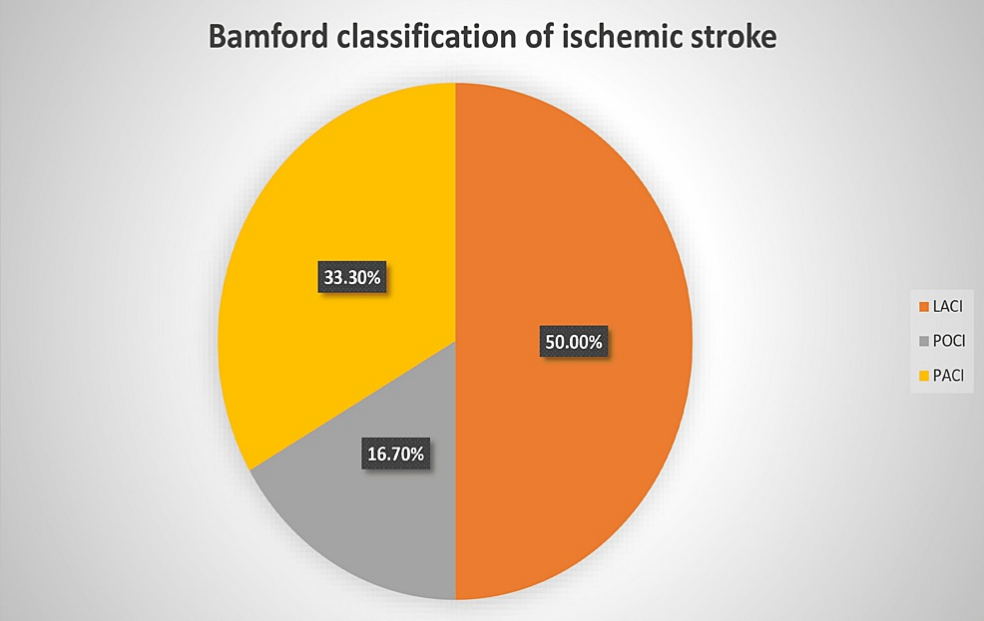
Types of ischemic stroke based on Bamford classification (n=30). LACI: lacunar anterior cerebral infarction; POCI: posterior circulation; PACI: partial anterior circulation.

Most of the cases (15, 50.0%) had lacunar anterior cerebral infarction (LACI), 10 (33.3%) involving partial anterior circulation (PACI), and five (16.7%) in the posterior circulation (POCI) (Figure 4).

**Figure 5:**
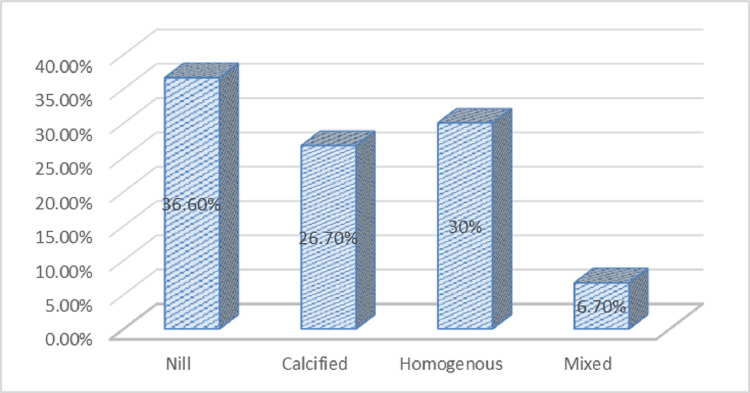
Types of plaques in ischemic stroke patients (n=30).

The characteristics of the carotid plaques were recorded. The majority of the ischemic stroke cases (30%) had homogenous plaques, followed by calcified (26.7%) and mixed (6.7%) plaques in the Doppler findings. About 36.6% of patients showed no plaque in the Doppler study (Figure 5).

The majority of ischemic stroke patients had an age >60 years (53.3%), followed by 51-60 years (36.7%). The mean age of the ischemic stroke cases was 63.33±10.79 years. There was no statistically significant difference between cases and controls regarding age (p>0.05). In the cases group, 76.7% of cases had hypertension (HTN), 56.7% DM (diabetes mellitus), 50.0% both HTN and DM, and 40.0% had a smoking habit. The smoking habit was significantly higher in cases (p=0.007). Mean serum cholesterol, triglycerides (TG), and low-density lipoprotein (LDL) levels were compared between cases and controls. No significant difference has been found between both groups regarding lipid profile (Table 1).

**Table 1:** Distribution of study population according to age, risk factors, and lipid profile in ischemic stroke patients (n=60). The p-value was determined by the chi-square test, the Mann-Whitney U test, and the independent samples t-test. DM: diabetes mellitus; HTN: hypertension; TG: triglyceride; LDL: low-density lipoprotein.

	Case (n=30) (%)	Control (n=30) (%)	p-value
≤40 years	1 (3.3%)	1 (3.3%)	0.702
41-50 years	2 (6.7%)	4 (13.3%)	
51-60 years	11 (36.7%)	13 (43.3%)	
>60 years	16 (53.3%)	12 (40.0%)	
Mean age (years)	63.33±10.79	58.93±8.70	0.087
HTN	23 (76.7%)	18 (60.0%)	0.165
DM	17 (56.7%)	13 (43.3%)	0.302
Both DM and HTN	15 (50.0%)	12 (40.0%)	0.436
Smoking habit	12 (40.0%)	3 (10.0%)	0.007
Serum cholesterol (mg/dL)	172.60±57.70	174.30±44.45	0.544
TG (mg/dL)	161.13±76.15	177.33±104.51	0.712
LDL (mg/dL)	110.23±50.52	108.33±41.98	0.965
HTN	23 (76.7%)	18 (60.0%)	0.165
DM	17 (56.7%)	13 (43.3%)	0.302
Both DM and HTN	15 (50.0%)	12 (40.0%)	0.436
Smoking habit	12 (40.0%)	3 (10.0%)	0.007
Serum cholesterol (mg/dL)	172.60±57.70	174.30±44.45	0.544
TG (mg/dL)	161.13±76.15	177.33±104.51	0.712
LDL (mg/dL)	110.23±50.52	108.33±41.98	0.965

Table 2 shows that 60% of cases had an infarct on the left side, followed by 40.0% on the right side. About 56.7% were subcortical infarct, while 40.0% were cortical.

**Table 2:** CT/MRI of the brain findings in ischemic stroke patients (n=30). CT: computed tomography.

CT/MRI findings		Frequency (n)	Percentage
Side of infarct	Right	12	40
	Left	18	60
Region affected	Cortical	12	40
	Subcortical	17	56.7
	Both	1	3.3

Table 3 shows that the mean right CIMT was 0.79±0.10 mm and the mean left CIMT was 0.90±0.17 mm in the case group. Besides, the mean right CIMT was 0.62±0.18 mm and the mean left CIMT was 0.66±0.20 mm in the control group. CIMT was significantly higher in the cases group (p<0.001). The mean CPS was larger among cases in the right and left internal and left common carotid arteries, but it was not statistically significant (p>0.05). The mean values of the RI were higher in all the arteries among cases than in controls, and it was statistically significant in the left CCA (p<0.05). The mean values of the PI were higher in all the arteries among cases than in controls, but it was not statistically significant (p>0.05) (Table 3).

**Table 3:** Comparison of CIMT, CPS, RI, and PI among different groups (n=60). The p-value was determined by the Mann-Whitney U test and the independent samples t-test. CPS: carotid plaque size; CIMT: carotid intima-media thickness; CCA: common carotid artery; ICA: internal carotid artery; RI: resistive index; PI: pulsatility index.

	Region	Case (n=30), mean±SD	Control (n=30), mean±SD	p-value
CIMT (mm)	Right CIMT	0.79±0.10	0.62±0.18	0.000
	Left CIMT	0.90±0.17	0.66±0.20	0.000
CPS (mm)	CCA			
	Right	2.05±0.85 (n=6)	3.3±2.4 (n=2)	0.643
	Left	2.03±0.85 (n=2)	1.37±0.21 (n=3)	0.256
	ICA			
	Right	2.18±1.04 (n=13)	1.95±0.26 (n=6)	0.831
	Left	2.43±0.91 (n=11)	1.62±0.66 (n=6)	0.075
RI	CCA			
	Right	0.79±0.06	0.76±0.06	0.054
	Left	0.78±0.07	0.74±0.06	0.043
	ICA			
	Right	0.68±0.08	0.66±0.07	0.236
	Left	0.67±0.10	0.64±0.07	0.192
PI	CCA			
	Right	1.76±0.29	1.62±0.27	0.092
	Left	1.69±0.32	1.53±0.27	0.055
	ICA			
	Right	1.31±0.28	1.23±0.21	0.230
	Left	1.27±0.46	1.16±0.22	0.295

We have tried to see the associations of CIMT, RI, and PI with different variables like the involved regions of the brain, age groups, diabetes, blood pressure, and smoking status. The cases with age more than 60 years had left ICA RI and PI values significantly higher than the cases who were younger than 60 years (p<0.05). We have also found a significant association between smoking history and left CCA RI ad PI values (p<0.05). However, we did not find any other significant associations among other variables (p>0.05) (Table 4).

**Table 4:** Comparison and association of CIMT, RI, and PI among the participants in different subgroups of acute ischemic stroke (n=60). The p-value was determined by the Mann-Whitney U test. CIMT: carotid intima-media thickness; CCA: common carotid artery; ICA: internal carotid artery; RI: resistive index; PI: pulsatility index; DM: diabetes mellitus.

		Region of brain			Age			Diabetes			Blood pressure			Smoking history		
Parameters	Region	Cortical (n=12), mean±SD	Subcortical (n=17), mean±SD	p- value	Age ≤60 years (n=14), mean±SD	Age >60 years (n=16), mean±SD	p-value	DM (n=17), mean±SD	Non-DM (n=13), mean±SD	p- value	Hypertensive (n=23), mean±SD	Non-hypertensive (n=7), mean±SD	p- value	Smoker (n=12), mean±SD	Non-smoker (n=18), mean±SD	p-value
CIMT	Right	0.82±0.08	0.77±0.10	0.263	0.78±0.09	0.79±0.11	0.728	0.78±0.10	0.80±0.09	0.711	0.78±0.09	0.81±0.11	0.501	0.80±0.09	0.78±0.10	0.465
	Left	0.89±0.19	0.92±0.17	0.711	0.92±0.18	0.88±0.17	0.473	0.90±0.17	0.91±0.18	1.000	0.92±0.16	0.82±0.21	0.266	0.87±0.16	0.92±0.18	0.391
RI	CCA															
	Right	0.78±0.05	0.80±0.07	0.499	0.78±0.05	0.81±0.06	0.224	0.78±0.06	0.81±0.06	0.263	0.79±0.05	0.78±0.08	0.564	0.78±0.03	0.80±0.07	0.573
	Left	0.78±0.05	0.78±0.07	0.679	0.76±0.03	0.78±0.08	0.334	0.77±0.07	0.78±0.07	0.592	0.77±0.06	0.79±0.08	0.501	0.74±0.04	0.79±0.07	0.043
	ICA															
	Right	0.67±0.06	0.69±0.08	0.845	0.66±0.06	0.69±0.08	0.313	0.67±0.08	0.70±0.07	0.432	0.68±0.07	0.67±0.10	0.962	0.67±0.05	0.68±0.09	0.950
	Left	0.67±0.11	0.68±0.09	0.853	0.63±0.07	0.70±0.10	0.004	0.68±0.11	0.67±0.08	0.812	0.67±0.10	0.67±0.10	0.940	0.64±0.06	0.69±0.11	0.276
PI	CCA															
	Right	1.71±0.24	1.80±0.33	0.616	1.67±0.22	1.83±0.33	0.240	1.71±0.28	1.82±0.31	0.341	1.77±0.26	1.73±0.40	0.532	1.68±0.13	1.81±0.36	0.573
	Left	1.70±0.25	1.70±0.36	0.586	1.60±0.14	1.75±0.41	0.355	1.66±0.33	1.72±0.31	0.563	1.65±0.29	1.77±0.41	0.471	1.54±0.18	1.78±0.36	0.039
	ICA															
	Right	1.27±0.21	1.36±0.32	0.879	1.23±0.21	1.37±0.32	0.294	1.26±0.27	1.37±0.29	0.408	1.31±0.27	1.28±0.33	1.000	1.27±0.17	1.33±0.33	0.950
	Left	1.30±0.50	1.32±0.36	0.926	1.14±0.27	1.36±0.6	0.013	1.26±0.57	1.27±0.31	0.650	1.26±0.49	1.28±0.38	0.901	1.07±0.38	1.38±0.48	0.173

## Discussion

Atherosclerosis is the main pathological factor in the majority of ischemic stroke cases. An acute ischemic stroke patient's CPS, CIMT, RI, PI, and other risk variables were studied to see if they were related.

In our study, among 30 cases, the majority of respondents were >60 years of age (53.3%). The mean age of the cases and controls was 63.33±10.79 years and 58.93±8.70 years, respectively. Age is the most common non-modifiable risk factor for the development of stroke. In this study, males accounted for 73.3% of ischemic stroke cases, while females accounted for only 26.7%. The male-to-female ratio was 2.75:1. In a previous large-scale study on Bangladeshi patients, the mean age of the stroke was 60.6 years, and 68% of the cases were male [17]. So, our findings were similar to the previous study done on the same population. Moreover, as the current study was done in a military hospital where most of the patients were veterans, the frequency of male patients was likely to be more than female patients.

We have compared the frequency of HTN, DM, and smoking status among cases and controls. All these three risk factors are strongly associated with ischemic stroke [18]. Surprisingly, in our study, only the smoking habit was significantly higher in cases (p=0.007). Maybe the smaller sample size in our study was the reason behind such findings.

As per the Bamford classification of ischemic stroke, half of the (50.0%) cases had LACI, which was also the commonest subtype in previous studies [19,20].

In the present study, the CIMT was significantly higher in patients with acute ischemic stroke when compared to controls. The mean right CIMT was 0.79±0.10 mm and the mean left CIMT was 0.90±0.17 mm among cases, which is similar to the values reported in another Indian study [21] but lower than the reported values from developed countries [22,23]. In the Italian study by Cupini et al., the mean CIMT was 1.04 mm in patients with non-lacunar stroke and 0.91 mm in patients with lacunar stroke [22].

The mean right CIMT and left CIMT for diabetics among cases were 0.73±0.16 mm and 0.81±0.20 mm, respectively. This observation was comparable with a previous publication where the mean CIMT was found to be 0.88±0.22 in DM [24]. Overall, our cases had lower CIMT values compared to other studies. As most of the cases were military veterans maintaining an active lifestyle, which may contribute to their lower CIMT values. There may be ethnic variation also, as the CIMT values were higher in the studies done on the Western population [22,23]. Systemic reviews also showed that Asians have lower mean CIMT values than other ethnic groups [25]. 

In the study, among 30 cases, 63.4% showed plaques on the Doppler, and the remaining 36.6% did not show on the Doppler. Among that 63.4%, 30.0% were homogenous, 26.7% calcified, and 6.7% mixed. The mean CPS was larger among cases in the right and left internal, external, and common carotid arteries except for the right CCA, but it was not significant statistically. There is increasing evidence that active unstable plaques in the carotid arteries are more prone to embolization, regardless of the degree of stenosis [26].

The mean RI and PI among cases in all the arteries were more than the controls; however, RI was statistically significant in the left CCA only. RI and PI were described as potential hemodynamic factors in stroke patients in other previous studies [13,27].

The CIMT, RI, and PI values among the DM patients of cases showed no significant difference from the non-DM cases. However, a significant association between carotid Doppler ultrasonography variables and diabetes mellitus was found in some previous studies [24,28]. We have found significantly higher values of RI and PI in the left ICA of the older patients. The association between aging and carotid atherosclerosis was also found in previous studies [6,27,28]. Smoking history was found to have a significant association with left CCA RI and PI values. Smoking had a significant impact on carotid duplex parameters in other previous studies [6,27]. Our study showed that these atherosclerosis markers are not associated with HTN, which is contrary to previous studies [6,27]. Patients with cortical infarcts may have more severe carotid atherosclerotic disease than patients with subcortical infarcts [29]. However, we did not find any association between the infarct locations and the parameters of carotid atherosclerosis. 

Most of the parameters of carotid atherosclerosis were found to be statistically significant on the left side only. The left carotid artery is more vulnerable to atherosclerotic changes compared to the right side, as it is a direct branch from the aorta and is under higher arterial pressures. Unilateral atherosclerosis is more common on the left side, and plaque thickness is greater on the left side. Also, the plaque characters are more vulnerable on the left side [30]. 

This study has some limitations. The sample size was small. Due to the purposive sampling technique, the case selection process was prone to researcher bias. We did not compare the carotid USG parameters between the symptomatic and asymptomatic sides. As the study was done in a military hospital, most of the patients were veterans, who may not represent the general population. As fitness is a mandatory requirement for military service, military persons lead an active life even after retirement. They may have less severe cardiovascular risk factors, including carotid atherosclerosis, compared to civilians. A large comparative study may be done to see whether any differences in these variables are present between military persons and civilians.

## Conclusions

The mean CIMT was significantly thicker among the patients with ischemic stroke compared to the control group. RI was found significantly more in the left CCA of the cases than in the controls. Our study showed age and smoking habit had an association with carotid atherosclerosis. We have found asymmetry of carotid atherosclerosis, more significant on the left side. Further large-scale studies will help to conclude the association of carotid duplex study findings with acute ischemic stroke patients in the Bangladeshi population.
